# Nasopharyngeal carcinoma with leptomeningeal metastases has been treated with comprehensive treatment for long-term survival: A case report and literature review

**DOI:** 10.1097/MD.0000000000037853

**Published:** 2024-06-07

**Authors:** Yi Yang, Jiajia Jiang, Yajing Liu, Shuanghao Feng, Hui Bu

**Affiliations:** a Department of Neurology, The Second Hospital of Hebei Medical University, Shijiazhuang, Hebei, China.

**Keywords:** immunotherapy, intrathecal chemotherapy, leptomeningeal metastasis, nasopharyngeal carcinoma, targeted therapy

## Abstract

**Rationale:**

Nasopharyngeal carcinoma has a high incidence in East and Southeast Asia, often with distant metastasis. However, leptomeningeal metastasis (LM) is extremely rare and usually has a poor prognosis. This paper reports the clinical treatment of a patient with meningeal metastasis of nasopharyngeal carcinoma (NPC) in order to improve the clinician’s understanding of the disease. Early diagnosis of the disease can alleviate the pain of patients and prolong their survival time.

**Patient concerns:**

We report the case of a 55-year-old female with a history of NPC with LM. Brain magnetic resonance imaging showed temporal lobe enhancement, peripheral edema, and enhancement of the adjacent meninges. Cerebrospinal fluid cytology suggests the presence of malignant tumor cells.

**Diagnoses:**

The patient was diagnosed with LM from NPC.

**Interventions:**

The patients were regularly given targeted therapy with nimotuzumab, immunotherapy with karyolizumab, and lumbar intrathecal methotrexate chemotherapy and supportive treatment.

**Outcomes:**

The patient had survived for 3 years since the diagnosis of LM and was in good condition and still under active antitumor treatment.

**Lessons:**

Leptomeningeal metastasis of NPC is a rare disease. Although there is currently no unified treatment plan, the neurological symptoms can still be controlled and the quality of life can be improved through active treatment.

## 1. Introduction

Leptomeningeal metastasis (LM) broadly refers to the disease caused by the metastasis of various malignant tumors to the leptomeninges and subarachnoid space.^[1]^ LM diagnosed clinically occurs in 5% of patients with solid tumors, and any cancer can metastasize to the leptomeninges,^[2]^ with lung cancer, breast cancer, gastric cancer, and melanoma as the main cancers. However, leptomeningeal metastasis of nasopharyngeal carcinoma (NPC) is extremely rare, and only a few isolated cases have been reported in the literature. Given the rarity of this presentation in NPC and the lack of clear standard treatment guidelines, we report this case in the hope of providing valuable insight into clinical diagnosis and treatment.

## 2. Case report

In November 2018, a 55-year-old female with nasal obstruction and hearing loss for 3 months was diagnosed with NPC (differentiated non-keratinizing squamous cell carcinoma) without significant evidence of distant metastasis. From December 2018 to February 2019, the patient received 60 Gy in 30 complete radiotherapy, and from March 2019 to July 2019, he received paclitaxel 210 mg + carboplatin 40 mg chemotherapy regimen for 2 cycles, receiving nimotuzumab 200 mg once a week for 6 weeks. After treatment, the patient did not complain of discomfort for 1 consecutive year.

In December 2020, she developed numbness, distension, and dizziness in both sides of the face and both upper limbs. The computed tomography (CT) scan of her head performed in the local hospital only showed multiple ischemic lesions in the brain, which was clinically diagnosed as “cerebral infarction,” and no response was seen after treatment. After half a month, the patient experienced frequent headache and memory decline.

**Figure 1.**
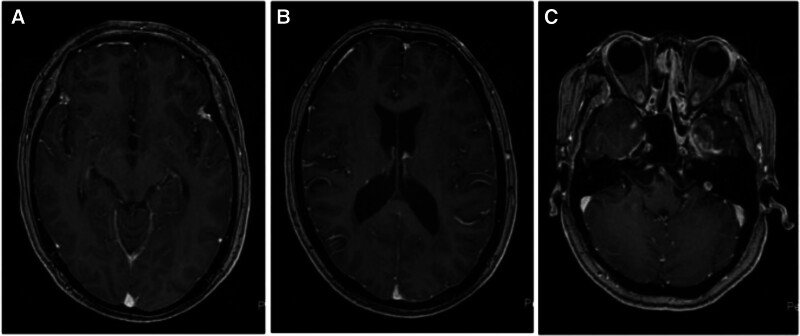
MRI of the patient’s brain at the time of confirmed leptomeningeal metastases: (A, B) meningeal enhancement, (C) left temporal lobe, and peripheral edema. MRI = magnetic resonance imaging.

**Figure 2.**
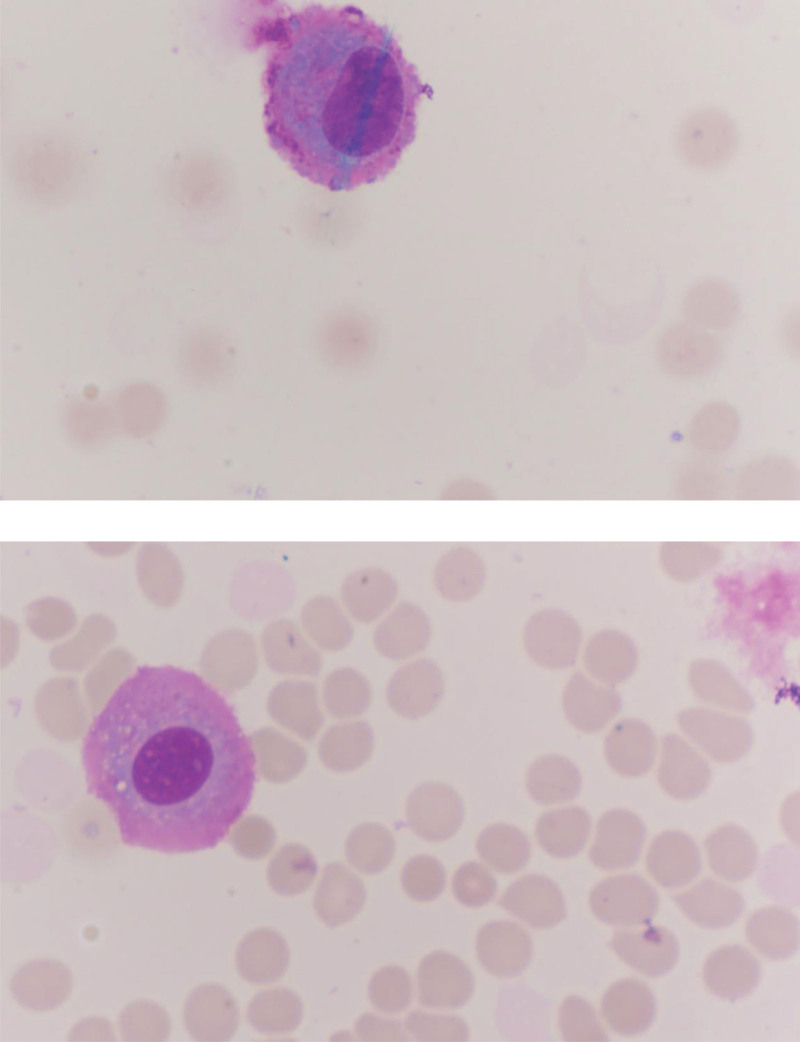
Cytological examination of CSF in a patient with confirmed leptomeningeal metastasis (MGG staining ×1000): 2 tumor cells were visible. CSF = cerebrospinal fluid.

The patient was then immediately transferred to our hospital. Neurological examination showed decreased orientation and memory, and positive neck resistance. Further brain MRI showed lobar enhancement and peripheral edema in the left temporal lobe adjacent to meningeal enhancement (Fig. 1). As our patient had a history of tumor and clinical manifestations of increased intracranial pressure, we performed a lumbar puncture to identify the cause, and her CSF pressure was elevated at 270 mm H_2_O (normal 80–180 mm H_2_O), white blood cells at 4 × 10^6^/L (normal 0–8 × 106/L), glucose at 5.44 mmol/L (normal 2.5–4.5 mmol/L) and protein at 0.67 g/L (normal 0.15–0.45 g/L), and CSF cytology showed 2 tumor cells (Fig. 2). Finally, based on the above findings, the patient was diagnosed with LM from nasopharyngeal carcinoma.

**Figure 3.**
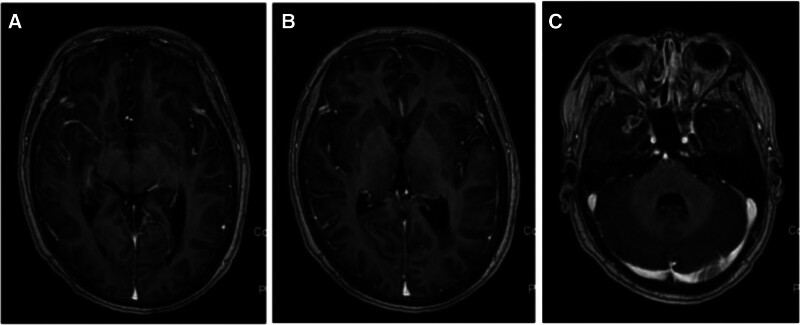
Follow-up visit in October 2023. Compared with the confirmed diagnosis, the patient’s brain MRI showed no significant deterioration. The left temporal lobe enhancement disappeared, and new right temporal lobe enhancement was found. MRI = magnetic resonance imaging.

**Figure 4.**
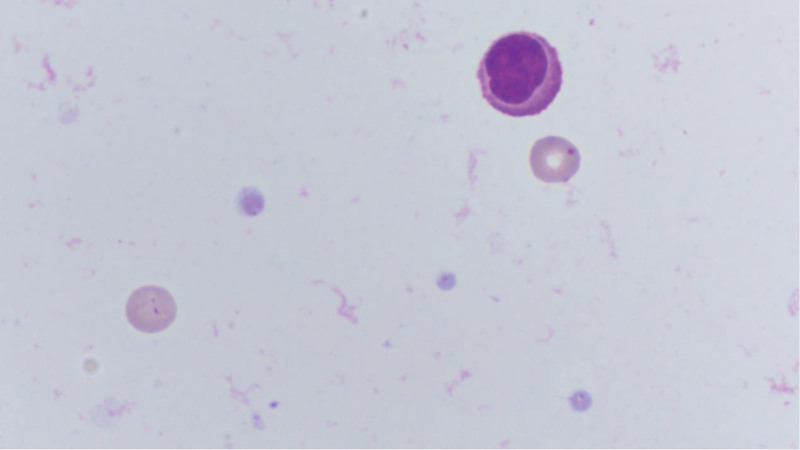
Follow-up visit in October 2023. Lymphocytes, monocytes, and erythrocytes were observed in CSF cytology of the patient. CSF = cerebrospinal fluid.

After the diagnosis was confirmed, our patient was given 200 mg karleilizumab every 21 days for a total of 6 times, and lumbar intrathecal injection of the chemotherapy drug methotrexate 10 mg + dexamethasone 5 mg for a total of 4 courses, and supportive treatment was given. The patient improved significantly after treatment. From 2021 to 2023, the patient came to our hospital regularly for lumbar intrathecal chemotherapy with methotrexate and received a second course of nimotuzumab. In the follow-up visit in October 2023, the brain MRI reexamined by the patient showed no obvious deterioration compared with that at the time of definite diagnosis, and the enhancement of the left temporal lobe disappeared, except for the new enhancement of the right temporal lobe (Fig. 3). The pressure value of CSF obtained through lumbar puncture was within the normal range, together with proteins and glucose. Cytology of CSF showed lymphocytes, monocytes, and erythrocytes (Fig. 4), and no tumor cells were found. Up to now, the patient is still alive without obvious deterioration. We will continue to pay attention to the changes of MRI and cerebrospinal fluid (CSF).

## 3. Discussion

NPC is one of the common malignant tumors in the head and neck region, and most commonly occurs in East and Southeast Asia.^[3]^ At present, it is considered that NPC has familial aggregation, which can involve 3 generations and is related to Epstein–Barr virus,^[4]^ environment and other factors. Common clinical symptoms include bloody nasal discharge, feeling of ear blockage, hearing loss, cervical lymph node enlargement, and cranial nerve symptoms. With the continuous improvement of radiotherapy and treatment options, the long-term survival rate of patients with NPC has been greatly improved. However, distant metastasis is still the reason for the treatment failure of nasopharyngeal carcinoma. The typical distant metastasis sites are liver, lung and bone. In clinical practice, metastasis of NPC to leptomeninges is extremely rare. However, once it occurs, the prognosis is extremely poor.

LM is a fatal disease, usually manifested as increased intracranial pressure, nausea and vomiting, meningeal irritation sign.^[5]^ For patients with malignant tumor combined with typical clinical symptoms, brain MRI can be used as the diagnosis basis of LM, with the sensitivity of about 70% to 85% and specificity of about 75% to 90%. The combination of brain MRI and CSF cytology can improve the diagnosis rate of LM. The median overall survival after the diagnosis of LM is only 4 to 6 weeks, while it may be extended to 10 to 15 weeks in patients receiving treatment.^[6]^ Slightly longer survival can be achieved only in patients with breast cancer or malignant tumors of the lymphatic system. Therefore, our goal in the treatment of LM is to improve the patient’s quality of life and prolong the patient’s survival. EANO-ESMO guidelines recommend multidisciplinary treatment for such patients.^[7]^ Therapeutic methods include intrathecal and systemic chemotherapy, radiotherapy, targeted therapy, immunotherapy, surgical treatment, and necessary supportive treatment.

Due to the special anatomical structure and anatomic site of nasopharynx, NPC is prone to invasion of adjacent structures, mainly involving the cervical lymph nodes. Therefore, the recognized and effective method for the treatment of nasopharyngeal carcinoma is radiotherapy, or comprehensive treatment mainly involving radiotherapy.^[8]^ Radiotherapy techniques include fixed field intensity-modulated radiotherapy, volume rotation intensity-modulated radiotherapy and spiral tomotherapy. Adverse reactions of radiotherapy include dry mouth, cranial nerve injury, brain or spinal cord radiation injury, radioactive bone necrosis.

In recent years, targeted drugs with strong specificity have gradually entered people’s field of view. Some molecules, such as epidermal growth factor receptor (EGFR) and vascular endothelial growth factor (VEGF), are highly expressed in NPC. EGFR is a tyrosine kinase receptor. EGFR signaling pathway plays an important role in DNA synthesis, cell growth and survival. In NPC patients, the overexpression of EGFR reaches more than 30%, which is related to tumor metastasis and prognosis.^[9]^ Nimotuzumab is a humanized monoclonal antibody targeting EGFR, which can specifically block EGFR and increase the sensitivity of tumor cells to radiotherapy. Due to its high selectivity, it also reduces the occurrence of many adverse reactions. At the same time, cetuximab can also be combined with chemotherapy in the treatment of advanced NPC, and it can achieve 100% short-term complete remission rate, but the adverse reaction is higher.^[10]^ As early as in 1971, Folkman^[11]^ believed that the growth, proliferation and metastasis of tumors depended on angiogenesis, and proposed a new view: tumor growth and proliferation could be inhibited by inhibiting tumor angiogenesis, of which VEGF was the most important angiogenic factor. Recombinant human endostatin, as a broad-spectrum targeted antiangiogenic drug, has been widely used in clinical practice to induce apoptosis of vascular endothelial cells and inhibit tumor growth.

At the same time, immunotherapy has brought new hope to patients with NPC. Under physiological conditions, the combination of programmed death-1 (PD-1) and programmed death-ligand 1 (PD-L1) as a common inhibitory signal mediating T cell activation inhibits the killing ability of T cells, thus preventing the occurrence of immune-related diseases. Most tumor cells express PD-1 and can combine with PD-L1 to exert an inhibitory effect on the activation of lymphocytes, thereby promoting the immune escape of tumors.^[12]^ Carleilizumab is a PD-1 inhibitor. In the second-line treatment study of metastatic NPC with carleilizumab, the objective response rate (ORR) reached 34% and the median progression-free survival (PFS) was 5.6 months, carried out by Fang et al,^[13]^ proving that carleilizumab is a potential treatment option with good tolerance for patients with recurrent or metastatic NPC.

Intrathecal chemotherapy refers to the direct injection of antitumor drugs into CSF by lumbar puncture and other methods, in order to avoid the blood–CSF barrier, increase the exposure of drugs in CSF, and reduce the systemic toxicity. Commonly used chemotherapy drugs for intrathecal injection include methotrexate, cytarabine, and cefoxitin.^[14]^

After the initial diagnosis of NPC in our patient, radiotherapy, chemotherapy, and nimotuzumab therapy were used, and the tumor was temporarily controlled. One year later, the patient’s condition worsened, characterized by headache, dizziness, and numbness of the limbs. The cranial MRI showed meningeal enhancement with edema, which supported the clinical judgment of meningeal involvement. The CSF cytology showed malignant tumor cells. In combination with the previous history of nasopharyngeal carcinoma, the LM of NPC could be definitely diagnosed. Since the diagnosis, the patient has been actively treated with carleilizumab, intrathecal chemotherapy and the second course of nimotuzumab therapy. Currently, the patient is still alive with a high quality of life.

This is a retrospective study. The patient underwent biopsy in another hospital, so there is no initial pathological data. The patient has low compliance and irregular follow-up visits. Recent studies have shown that immunotherapy is beneficial for patients with leptomeningeal metastasis: treatment with immune checkpoint inhibitors caused significant changes in the characteristics of immune cells in the CSF of patients. However, the patient reported in this paper only received 2 courses of nimotuzumab, so the long-term efficacy of immunotherapy cannot be observed.

## 4. Conclusion

In conclusion, LM of NPC is rare. As the survival time after diagnosis is usually short, early diagnosis is crucial. The survival time of patients can be improved by means of brain MRI and CSF cytology together with corresponding active treatment.

## Acknowledgments

We thank other members of the Department of Neurology of The Second Hospital of Hebei Medical University for their critical comments.

## Author contributions

**Writing – original draft:** Yi Yang.

**Writing – review & editing:** Yi Yang, Hui Bu.

**Conceptualization:** Jiajia Jiang.

**Resources:** Yajing Liu.

**Validation:** Shuanghao Feng.
